# Turnaround time and barriers to treatment of newly diagnosed cancer in Uganda: a mixed-methods longitudinal study

**DOI:** 10.4314/ahs.v22i1.40

**Published:** 2022-03

**Authors:** Solomon Kibudde, Eve Namisango, Annet Nakaganda, Mackuline Atieno, Joy Bbaale, Martin Nabwana, Fatia Kiyange, Meg O'brien, Emmanuel BK Luyirika, Jackson Orem

**Affiliations:** 1 Uganda Cancer Institute, Kampala, Uganda; 2 African Palliative Care Association, Entebbe, Uganda; 3 Cicely Saunders Institute, King's College London; 4 School of Public Health, Makerere University College of Health Sciences

**Keywords:** Turnaround time, steps, barriers, waiting time, cancer, Uganda

## Abstract

**Introduction:**

Cancer represents a growing public health concern. Late-stage at diagnosis, limited access to effective treatment, and loss to follow-up are responsible for dismal outcomes.

**Objective:**

To describe care pathways, turnaround times, and identify barriers to timely initiation of cancer treatment

**Methods:**

Using a sequential mixed-methods design involving focus group discussions, we followed up 50 participants between January, and June 2018. We computed the median observed turnaround time to treatment (TTT) at each care step and reported delay as deviations from the proposed ideal turnaround times.

**Results:**

The ideal TTT with either chemotherapy, or radiotherapy, or surgery was 8, 14, and 21 days respectively. At a median follow-up time of 35.5 days (IQR 17–66), only 29 of the 50 study participants had completed all steps between registration and initiation of treatment, and the observed median TTT was 16 days (9 – 22 days) for chemotherapy, and 30 days (17 – 49 days) for radiotherapy, reflecting a significant delay (p-value = 0.017). Reported barriers were; shortage of specialists, patients required visits to outside facilities for staging investigations, prohibitive costs, poor navigation system and time wastage.

**Conclusions:**

When compared to the recommended ideal turnaround time, there was significant institutional delay in access to chemotherapy and radiotherapy attributed to multiple external and internal healthcare system barriers.

## Introduction

Cancer burden in sub-Saharan Africa represents a major growing public health concern. Late-stage at diagnosis, limited access to effective treatment, and loss to follow-up are largely responsible for the dismal outcomes^1^. Access to care is dependent on health systems, and patient's social context. Health system barriers to cancer treatment in sub-Saharan Africa include; few radiotherapy infrastructures^2^, poor availability of chemotherapy and palliative care 3,4, shortage of oncologists 4,5, and limited capacity to deliver cancer surgery 6. While patient-level factors include high out of pocket expenditure on treatment cost 5,7,8, and insufficient use of resources 1.

Although several studies have investigated barriers to accessing cancer treatment in Africa, these have focused largely on specific cancer types namely cervix cancer^7^,^9^,^10^ and Burkitt's lymphoma^11^. Notably, cancer treatment involves different treatment modalities such as chemotherapy, radiotherapy, surgery, and immunotherapy. There is scanty information on barriers to access to comprehensive cancer care involving multiple treatment modalities as faced by patients in the real-world clinical setting. In addition, few of these studies have investigated health system barriers within cancer referral centres^9^. Prolonged waiting time is associated with a negative impact on cancer survival, and institutional delay is reported to contribute significantly to overall treatment delay.

In patients with newly confirmed cancer diagnosis referred for care, access pathways, turnaround times, and barriers to timely cancer treatment remain largely unknown. Exploring steps and barriers to care within a cancer treatment facility is envisaged to facilitate the identification of priority areas for quality improvement and optimal resource utilization. At the Uganda Cancer Institute (UCI), the cancer care pathway from registration to initiation of treatment involves several discreet steps. This study aimed to clearly map out these steps, estimate the ideal and measure the actual turnaround times for each step, and identify barriers that delay each step that in sum, delay timely access to cancer treatment at the UCI.

## Methods

### Design

We used a sequential mixed-methods approach. In phase I, we conducted exploratory focus group discussions (FGDs) with oncology health care workers (HCWs) to map out the ideal steps and turnaround time of access to routine cancer care within a cancer centre. This was followed by Phase II that involved a longitudinal follow-up of fifty participants between January 24, 2018, and June 30, 2018, to determine their respective turnaround times at the different steps of the care pathway between registration and treatment initiation. We also conducted two exit-FGDs with patients after treatment initiation to explore their experiences.

### Setting

The UCI is a public national cancer referral hospital in Uganda, with an estimated 4,000 new cancer patients annually. The institute provides a range of cancer treatment services including systemic chemotherapy, radiotherapy, surgery, and immunotherapy.

### Participants

The study population included cancer patients and key oncology healthcare workers. Eligible patient participants were required to have a histological cancer diagnosis, no prior oncologic treatment, age 18 years and above, and the ability to give informed consent in English and/or Luganda. We excluded inpatients and those with a poor performance status (Eastern Cooperative Oncology Group, ECOG score ≥ 3 i.e. patients confined to bed or chair more than 50% of waking hours, and only capable of limited self-care).

### Sample size and sampling procedure

Since no hypothesis was being tested in this study, no sample size computation was undertaken. Fifty participants were enrolled in a longitudinal cohort as recommended that the sample of fifty were sufficient for theme saturation for barriers to service access 12. All sampled patients were enrolled if they agreed to the study procedures and signed written informed consent. Participants were numbered sequentially, and every 4th entry patient was systematic sampled^13^ for screening; and were enrolled if they agreed to study procedures. For participants in FGDs, purposive sampling was used to select oncology healthcare workers with extensive experience regarding cancer care steps.

### Procedures and data collection

**Phase I:** Before patient recruitment, three investigators conducted two FGDs with oncology healthcare workers. The findings from this assessment were used to prospectively explore turnaround time and perceived reasons for delay among the patient cohort.

**Phase II:** A pretested structured questionnaire was completed by a trained research assistant for each patient participant at four pre-specified study visits between registration, and initiation of cancer treatment. The primary outcome was turnaround time at each step, while secondary outcomes were overall turnaround time to treatment (TTT) defined as the time interval from registration at UCI to start of treatment; and waiting time to treatment (WTT), defined as the time interval from date of cancer diagnosis (prior referral to UCI) to date of initiation of cancer treatment. Two sex-stratified FGDs for patients were conducted to gain a deeper understanding of the system barriers. The discussion was audio-recorded and field notes were also taken and expanded within 24 hours.

### Statistical analysis

Quantitative data was entered using the EpiData version 3.1 software, cleaned and exported to Stata 15.0 for analysis. Continuous variables were summarized as means, medians, and range; while categorical data were presented as proportions. Turnaround time and intervals of care steps were computed from the start dates of each step. All audio and field notes were transcribed verbatim and imported into QSR NVivo version 12 for thematic analysis.

### Ethical considerations

Ethics approval was obtained from the Uganda Cancer Institute Research and Ethics Committee (UCIREC REF #: 01-2017), and the Uganda National Council for Science and Technology before any study procedure. Participation was voluntary and written informed consent was obtained before data collection. All the hardcopy and electronic data were decoded and kept with restricted access to ensure confidentiality. A transport refund fee was approved by the ethics committee and issued at every study visit.

## Results

**Figure 1:**
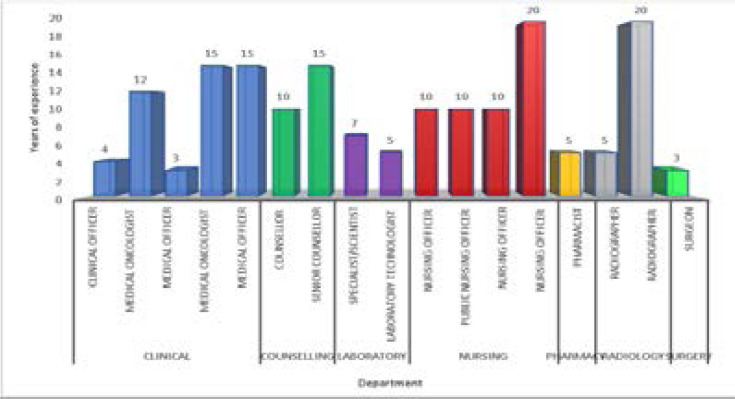
Profile of participants for the FGD with oncology healthcare workers

**Figure 2:**
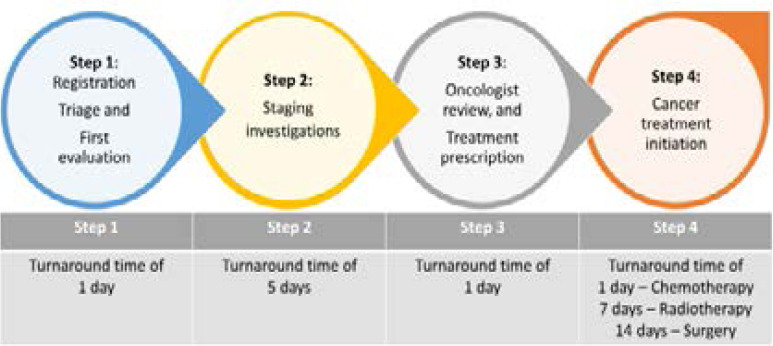
Stepwise processes outlining the cancer care pathway at the UCI and ideal turnaround times.

Cancer care pathway, steps and ideal turnaround time We conducted two FGDs with seventeen oncology healthcare workers (Figure 1) to understand the steps involved in the cancer care pathway. The pathway entailed a sequence of steps namely; registration, triage, and first evaluation (step 1), completing all staging investigations (step 2), review by oncologist and treatment prescription (step 3), and cancer treatment initiation (step 4) (Figure 2).

The proposed ideal turnaround times for care at steps 1, 2, and 3; were suggested as; 1, 5, and 1 day(s) respectively. While at step 4, initiation of chemotherapy, radiotherapy, and surgery were 1, 7, and 14 days respectively (Figure 2). Overall, the proposed ideal turnaround time to initiation of cancer treatment with either chemotherapy, or radiotherapy, or surgery was 8, 14, and 21 days respectively.

### Observed turnaround time to cancer treatment

The median follow-up of fifty participants enrolled between January and June 2018, was 35.5 days (IQR 17-66). The participants completing a pre-specified visit were 50, 40, 40 and 29 at step 1, step 2, step 3, and step 4 respectively. The 21 participants who did not start treatment within the follow-up period included all three patients who had been prescribed to receive surgery; hence surgery was not included in this analysis (Tables 2 and 3). Among the 50 enrolled patients, the median age was 47 years (IQR 33 – 56 years), 30 (60%) participants were female, and 46 (92%) participants reported attaining primary level education. Sixteen (53.3%) of the 30 female participants were referred for the treatment of cervical cancer, and seven (23.3%) were referred for treatment of breast cancer (Table 1).

**Table 2:** Observed turnaround time and waiting time to treatment in days

Characteristic		Type of treatment given	
		
	Overall n=29	Chemotherapy n=15	Radiotherapy n=14
Observed turnaround time[1],			
**Median (IQR)**	18.5 (14.5, 36.5)	16 (9, 22)	30 (17, 49)
**Mean (SD)**	31 (31.5)	19.6 (17.0)	44.2 (39.3)
**Range**	2–119	2–69	3–119
Waiting time to treatment[2],			
**Median (IQR)**	33 (22, 49.5)	27 (17, 40)	48.5 (30, 130)
**Mean (SD)**	70.7 (98.4)	45.5 (61.1)	106 (130.4)
**Range**	16–416	16–250	20–416

[1] TTT = time from registration to initiation of treatment

[2] WTT = time from outside diagnosis to initiation of treatment

**Table 1:** Baseline clinical characteristics of patients

Characteristic	Total, n (%)	Treatment status		P-value
		
		Initiated treatment, n (%)	Did not initiate treatment, n (%)	
**Age**				
Mean (SD)	45.2 (14.4)	46.8 (15.4)	43.1 (12.8)	0.380
Median (IQR)	47 (33–56)	46.5 (32.5–56.5)	47 (33–56)	0.869
**Gender**				0.726
Male	20 (40.0)	11 (37.9)	9 (42.9)	
Female	30 (60.0)	18 (62.1)	12 (57.1)	
**Duration since first symptom (Months)**				
Mean (SD)	20.8 (33.4)	21.2 (32.9)	20.3 (34.9)	0.175
Median (IQR)	12 (4–24)	12 (6, 24)	6 (3–12)	0.079
**Diagnosis**				0.120
Bone cancer	1 (2.0)	0 (0.0)	1 (4.8)	
Breast cancer	7 (14.0)	6 (20.7)	1 (4.8)	
Bladder cancer	2 (4.0)	2 (6.9)	0 (0.0)	
Cervical cancer	16 (32.0)	10 (34.5)	6 (28.6)	
Colorectal cancer	1 (2.0)	0 (0.0)	1 (4.8)	
Esophagus cancer	4 (8.0)	1 (3.4)	3 (14.3)	
GTN	1 (2.0)	0 (0.0)	1 (4.8)	
Head and neck cancer	5 (10.0)	4 (13.8)	1 (4.8)	
Kaposi's sarcoma	2 (4.0)	2 (6.9)	0 (0.0)	
Liver cancer	1 (2.0)	1 (3.4)	0 (0.0)	
Lymphoma	3 (6.0)	1 (3.4)	2 (9.5)	
Prostate cancer	2 (4.0)	1 (3.4)	1 (4.8)	
Sarcoma	5 (10.0)	1 (3.4)	4 (19.0)	
**Performance status as measured by ECOG**				0.724
PS 0 or 1	37 (74.0)	22 (75.9)	15 (71.4)	
PS 2 or 3	13 (26.0)	7 (24.1)	6 (28.6)	
**Use of herbs or traditional healer before** **attending UCI**				0.151
Yes	25 (50.0)	17 (58.6)	8 (38.1)	
No	24 (48.0)	11 (37.9)	13 (61.9)	
Not recorded	1 (2.0)	1 (3.4)	0 (0.0)	
**Follow-up time** (Days)				
Median (IQR)	35.5 (17–66)	50 (36–91)	17 (4–28)	**<0.001**
Lost to follow-up	8 (16.0)	0 (0.0)	8 (38.1)	**<0.001**

UCI – Uganda Cancer Institute, ECOG – Eastern Cooperative Oncology Group, IQR -Interquartile range, GTN – Gestational trophoblastic neoplasm

Overall, 29 of the 50 participants were able to start cancer treatment with chemotherapy (15 participants) or radiotherapy (14 participants), and 21 participants had not started any treatment by study completion. Overall, the observed turnaround time to treatment with chemotherapy was a median of 16 days (IQR 9 – 22 days), compared to 30 days (IQR 17 – 49 days) for radiotherapy (Table 2). The median waiting time to treatment (time interval from diagnosis to start of treatment) was 33 days (IQR 22 – 49 days), with the median waiting time to chemotherapy of 27 days (IQR 17 – 40 days) compared to 48.5 days (IQR 30 – 150 days) for radiotherapy (Table 2).

When compared to the estimated ideal turnaround time, the median turnaround times for chemotherapy were significantly shorter for steps 1 and 3 (less than 1 day, compared to an ideal turnaround time of 1 day), and they were significantly delayed for step 2 (14 days compared to an ideal of 5 days). For radiotherapy, step 1 was significantly shorter than the estimated ideal turnaround time (less than 1 day compared to the ideal turnaround time of 1 day), while step 3 (2 days compared to an ideal of 1 day) and step 4 (20 days, compared to an ideal of 7 days) were significantly delayed (Table 3).

**Table 3:** Comparison of ideal and observed turnaround time at each step of care

Step	N[*]	Ideal turnaround time	Observed turnaround time				
			Overall	Chemotherapy		Radiotherapy	
				Median (IQR)	p- value[†]	Median (IQR)	p-value[‡]
**Step** **1**	50	1 days	0 (0–1)	0 (0–1)	**0.009**	0 (0 – 1)	**0.041**
**Step** **2**	40	5 days	8.5 (6 – 15.5)	14 (7–20)	**0.003**	6 (4 – 8)	0.483
**Step** **3**	40	1 day	1 (0 – 2)	0 (0 – 1)	**0.011**	2 (1 – 5)	**0.042**
**Step** **4**	29	1 day - Chemotherapy 7 days - Radiotherapy	6 (0 – 21)	1 (0 – 2)	0.792	20 (7 – 43)	**0.017**

[*] N = number of patients who completed the respective step of care pathway

[†] P-value comparing the ideal and observed turnaround time to chemotherapy

[‡] P-value comparing the ideal and observed turnaround time to radiotherapy

Step 1: Registration, Triage and First evaluation

Step 2: Staging investigations

Step 3: Oncologist review, and treatment prescription

Step 4: Cancer treatment initiation

### Barriers and reasons for delays in access to cancer treatment

**Figure 3:**
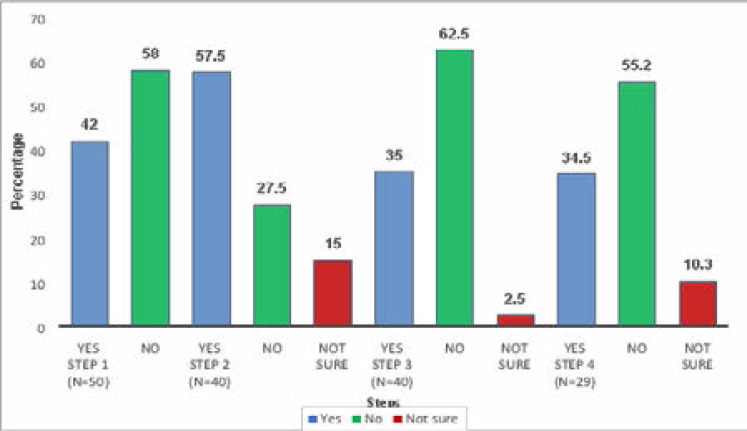
Timeliness at four steps of the care pathway Step 1: Registration, Triage and First evaluation Step 2: Staging investigations Step 3: Oncologist review, and treatment prescription Step 4: Cancer treatment initiation

Patients reported a lack of timeliness in care at all the four steps between registration and initiation of treatment (Figure 3). At step 1, 29 (58%) participants reported a lack of timeliness, and this was statistically significant when we compared the observed turnaround time to the ideal turnaround time (Table 3). Furthermore, nearly 28% of respondents in step 2 reported a lack of timeliness, and this correlated with a significant delay among patients treated with chemotherapy (p-value 0.003). At step 3, 62.5%of respondents reported lack of timeliness of their consultation with the oncologist (Figure 3), correlating with significant delay in the radiotherapy group (Table 3).

We correlated findings from the survey at every step of the care, with findings from FGDs with patients after initiation of cancer treatment. We found multidimensional barriers to timely access to cancer treatment crosscutting external factors (not controlled by the UCI) and internal factors (could be controlled by the UCI).

External factors contributing to delay between cancer diagnosis at the referral facility, and registration at the UCI included;

a) Stigma

Disease symptoms affected patients' willingness to interact with other patients and healthcare workers during care steps

“*Patients with offensive discharge related to cancer illness often hid outside of the waiting area*” Female FGD participant 04

“*While waiting for my appointment with the specialist, my condition became worse; and when I visited my local healthcare providers, they refused to treat me because I am a cancer patient*.” FGD Male participant 07

b) Use of alternative medicines

The tendency to use alternative medicines until noticeable failure before seeking known effective cancer treatment was reported and attributed to misinformation.

“*I was misled into believing that the herbs can cure cancer. I only came for treatment after noticing no improvement*” Male FGD participant 04

“*due to side effects associated with chemotherapy, and foul discharge from the tumour on the breast, I tried to seek alternative treatment hoping to reduce the smelling very fast*” Female study participant 35

c) Cost of transportation and accommodation

Outpatients receiving cancer treatment suffered financial burden associated with long-distance travel, coupled with high costs of renting temporary accommodation while awaiting and/or receiving cancer treatment. Some patients endured weeks to months lodging on verandas to minimize costs at the risk of other diseases including malaria.

“*I was given a series of tests to do and could not afford to travel back to my village between appointments. I stayed on the veranda for two weeks before starting cancer treatment. I suffered a malaria episode, became weaker and had to seek further care*” Female FGD participant 01

“*There was no accommodation, and so I slept outside on the hospital veranda as I awaited to start radiotherapy treatment*” Male study participant 04

d)Fear of cost of medical care (staging and treatment) at UCI

Patients decried high out of pocket expenditure of cancer medicines as a catalyst for poor compliance and delays. 12.5% of respondents reported purchasing cancer medicines at an outside pharmacy (Table 4, step 3).

**Table 4:** Patient's experiences with the timeliness of access to care

PROCESS	PATIENTS' EXPERIENCE AT CARE PROCESS	PROPORTION (%)
**STEP 1**[*] **(n = 50)**	Satisfactory	21 (42.0)
	Unsatisfactory (n=29, 58%)	
	Long queues at registration and clinician	18 (36)
	Delayed transfer of hospital chart to the clinician	6 (12)
	Lack of information on process/ navigation	1 (2)
	Time wastage/poor quality time with HCW	9 (18)

**STEP 2**[†] **(n = 40)**	Satisfactory	23 (57.5)
	Unsatisfactory (n = 11, 27.5%)	
	Long queues	5 (15.6)
	Delay of result processing	3 (9.4)
	Bribes before getting staging investigation	2 (6.3)
	Required to visit outside facility for a test	18 (56.3)
	Not sure	6 (15.0)

**STEP 3**[‡] **(n = 40)**	Satisfactory	14 (35.0)
	Unsatisfactory (n=25, 62.5%)	
	Oncologist was late	1 (6.25)
	Referral for a consultation to an outside hospital	1 (6.25)
	Delay due to ill health resulting in admission	2 (12.5)
	Purchase of cancer medicines	2 (12.5)
	Delay due to incomplete workup	8 (50)
	Not sure	1 (2.5)

**STEP 4**[§] **(n = 29)**	Satisfactory	10 (34.5)
	Unsatisfactory (n=16, 55.2%)	0
	Treatment delayed by the late arrival of HCW	3 (11.5)
	Long queues	8 (30.8)
	Delay of file transfer to the treatment room	2 (7.7)
	Bribes before receiving treatment	2 (7.7)
	Purchase of cancer medicines	2 (7.7)
	Delay due to inadequate workup	2 (7.7)
	Other delays	1 (3.8)
	Not sure	3 (10.3)

[*] Step 1: Registration, Triage and First evaluation

[†] Step 2: Staging investigations

[‡] Step 3: Oncologist review, and treatment prescription

[§] Step 4: Cancer treatment initiation

“*I nearly abandoned the treatment after failing to buy a prescribed cancer medicine estimated at over five hundred thousand Uganda Shillings (500,000 UGX)*” FGD Female participant 02

“*the doctor has prescribed this medicine for me four times and I do not buy it because it is very expensive. So, I decided to go home and die*.” FGD Female participant

“ *as a widow with three children, and no income at all, it is difficult to buy any medicines and yet they are very expensive*” study participant 09

After registration at the UCI, the following internal factors were report to contribute towards delay in care steps leading to initiation of treatment;

i. Inadequate staff (both medical specialists and other staff) leading to long queues

Patients expressed concern of a shortage of specialists resulting in long queues (36% at step 1, 15.6% at step 2 and 30.8% at step 4), occasionally rescheduling of visits, and longer turnaround time to treatment (Table 4). They remarked that cancer patients have complex needs and often require longer consultation time per patient.

“*The doctors are very few, yet the queue is long” and “each patient needs about 40 minutes with the doctor*” FGD Female participant 05

“*I underwent a CT scan but waited for 3 weeks to get the report. Later, the oncologist referred me to a private hospital for surgery, which I couldn't receive due to costs*” Study participant 08

Access to supportive treatment before and/or during definitive cancer treatment was hampered by long turnaround times.

“*I presented with severe symptoms and had to endure until the appointment with specialist*” Male FGD participant 02

“*While on treatment, I experienced difficulty in swallowing. I was given a doctors' appointment but had to stop the treatment because I could not feed*” Male FGD participant 07

ii. Poor appointment coordination (including patient folder misplacement)

Hospital patient-folder misplacement and/or loss resulted in missing appointment with the specialist, causing delay (12% at step 1, and 7.7% at step 4), time wastage (18% at step 1, and 11.5% at step 4), and consequently extra visits (Table 4).

“*My file was misplaced, I walked back and forth all day between the clinic and records office in vain. I cried terribly after the clinic staff told me to come back after 4 weeks*” Female FGD participant 03

“*When you come here following a referral, you just do not know where to start, and where to end. Even the investigations they tell us to do are scattered, the heart, the liver and many others*” FGD male participant 06

“*My treatment delayed as there were no directions on procedures after registration and thus, I was confused as to what to do since there were no patient navigators*” study participant 34

iii. Poor patient navigation to laboratory, radiology, pharmacy and other departments

Patients expressed difficulty in accessing service points like radiology, laboratory, pharmacy, etc.

“*I did not know where to find the laboratory for staging investigation, or the special test for my heart (cardiac echocardiography)*” Female FGD participant 04

The impact of structural navigation was worst felt by patients with advanced symptomatic disease.

“*Another patient was too weak to walk into the clinic waiting area; he waited at the veranda outside of the premises even though his number was called earlier*” Male FGD participant 06

“*Patients with very advanced disease who cannot queue up often waited longer until they found someone to assist them get close to the doctor*” FGD Male participant 06

iv. Doctor-patient communication breakdown

Feelings of hopelessness after learning about their cancer stage, and prognosis; in addition to limited access to information affected patients' coping response to pre-treatment procedures.

“*I requested for information on my illness and treatment steps but couldn't get it. This affected my interaction with that healthcare worker*” Male FGD participant 08

Furthermore, limited information and education about cancer, staging procedures, and its therapies including toxicities hindered crucial decision making resulting in treatment delays.

“*When I learnt about the side effects and pain that I was to endure while receiving chemotherapy and radiotherapy, I opted to wait until I experienced severe symptoms*” Female FGD participant 10

“*Some staff members are very rude; you request for clarification on certain things and they will respond by shouting. And, if you can't get help, you will go back home and when symptoms worsen, you come back and pray you find someone better.*” FGD Male participant 06

v. Cost of staging tests and treatments

Costs associated with staging investigations especially when patients had to pay out of pocket. 56.3% participants reported visiting an outside hospital to conduct a test privately (Table 4).

“*It took me over five weeks to get the investigations done due to associated costs; I struggled to raise over two hundred thousand Uganda shillings (200,000 UGX) that was required” “So I went back home until I found someone to help me. Government should help us fund some of these costs.*” Female FGD participant 06

## Discussion

Our findings suggest the recommended ideal turnaround time between registration and initiation of cancer treatment as 8, and 14 days for chemotherapy, and radiotherapy respectively. However, in this cohort, patients spent a median turnaround time of 16 days, and 30 days for chemotherapy and radiotherapy respectively. Notably, patients' experience with timeliness of care (Figure 3 and Table 4) was often different from the objective data in table 3. This highlights the fact that patients' perceptions may often be different from those of the healthcare workers. To the best of our knowledge, this study gives the first documentation of the care pathway, steps, and recommended timeframe at different steps before the initiation of cancer treatment in Uganda. The interval difference in turnaround time to chemotherapy as compared to radiotherapy relates to the additional radiotherapy planning processes including simulation, target delineation, dosimetry, and plan optimization. Our findings corroborate closely with two earlier studies at UCI albeit centred on access to chemotherapy. Buckle et al 11 investigated turnaround time to chemotherapy among children with endemic Burkitt's lymphoma and the median duration was 14 days, while Low et al 14 reported a median turnaround time of 15 days among adults with epidemic Kaposi's sarcoma. Notably, our cohort was heterogeneous with cervix cancer as the most frequent dignosis which requires a unique set of pre-treatment evaluation, as well as concurrent radiotherapy. We observed a significant delay to the start of radiotherapy (p-value 0.017). In patients with cervical squamous cell carcinoma and head and neck squamous cell carcinoma, delays in treatment could result in worse treatment outcomes. However, for breast and prostate cancer, this interval would less likely affect treatment outcomes in early stage disease.

We also found the overall waiting time for treatment (interval from cancer diagnosis to cancer treatment) as 33 days (range 16 – 416 days) with a median of 27 days for chemotherapy and 48.5 days for radiotherapy. Previous studies have demonstrated the negative impact of prolonged waiting time to treatment for certain cancers particularly squamous cell carcinomas of the head and neck region, and gynaecological cancers. Several studies have investigated delay in diagnosis of breast cancer 15,16, and Kaposi's sarcoma 17 in Uganda but few explored waiting times to treatment. Whereas an ideal interval time between diagnosis to treatment was not included in the scope of the FGDs with oncology HCWs, our findings correlate closely to published results from other countries in sub-Saharan Africa. In Ghana, among patients with breast cancer, the waiting time to treatment for breast cancer was 33 days 18, while in South Africa, patients with breast cancer waited for an average of 37 days 19. In our cohort, among three patients awaiting oncologic surgical treatment, none received surgery by end of study follow-up, and thus we couldn't assess waiting time to surgery as a treatment modality for cancer. This delay was over three months of follow-up, which represents a negative impact on treatment outcomes for certain cancers such as breast cancer. In a study among patients with breast cancer awaiting oncologic surgical treatment in South Africa, the treatment delay was 40 days as compared to 14 days for chemotherapy 19. In a recent survey at Uganda's national referral hospital among 430 women (15% among stage I-III), 18% of women with breast cancer had not initiated treatment within 1 year since diagnosis 20. And another study in 28 hospitals in Uganda showed that nearly 60% of breast cancer surgical procedures including biopsy were performed at the national referral hospital 6 confirming an earlier report that indicated significant challenges in the provision of safe, timely, and affordable surgical care in Uganda 4,21.

At all study visits, patients remarked a lack of timeliness, resulting in a significant delay at step 1, step 3, and step 4 (initiation of radiotherapy). The critical delay was at step 3 (Oncologist review and treatment prescription), and radiation therapy treatment; attributed to several reasons including; shortage of specialists resulting in long queues and appointment intervals, the prohibitive cost of treatment, a poor structural navigation system, and inefficient appointment coordination. This was consistent with reports from several studies evaluating delays in various cancer sites at treatment centres in sub-Saharan Africa 11,22–25. This cohort had ambulatory patients with a median duration of 12 months of symptoms at presentation to UCI, and indeed previous studies reported a high burden of late-stage disease at cancer diagnosis. In a study by Galukande et al 16, among 201 women with breast cancer, the median time from symptom onset to initiation of treatment was 12 months (range 1–120 months). While a recent survey among 162 women presenting for treatment of breast cancer at the national referral hospital, 84% had stage IV, while 10% had stage III disease 15. This contributes to patient distress which was reflected in the assessment of timeliness of cancer care steps. Two recent publications demonstrated the potential effect of attending HIV care and early referral for cancer treatment particularly for AIDS-associated malignancies 14,22. Our study confirmed a few under-reported barriers such as doctor-patient communication breakdown, stigma, and use ofalternative medicines 7.

Our results are not without limitations. Notably, we excluded inpatients and patients with poor performance status albeit they represent a small proportion; they are likely to have unique challenges in accessing cancer care. Secondly, multidisciplinary tumour boards were not incorporated since, at the time of data collection, there were few functional tumour boards. Other limitations included; small sample size, high rate of loss to follow-up (21 patients in this cohort did not start treatment) and poor representation from radiation oncology at the FGD with oncology healthcare providers. Despite our limitations, this study employed a robust study methodology incorporating a sequential mixed-methods design with the inclusion of FGDs, and no restrictions on cancer types.

## Conclusion

Although the recommended ideal waiting time between registration and initiation of cancer treatment at UCI was 8 days for chemotherapy, and 14 days for radiotherapy, patients waited slightly longer with a median of 16 days for chemotherapy, and 30 days for radiotherapy. We found a significant delay in turnaround time to both chemotherapy and radiation therapy treatment and contributing factors were: the shortage of specialists resulting inong queues and appointment intervals, the prohibitive cost of treatment, required visits to outside facilities for staging investigations, a poor structural navigation system, and inefficient appointment coordination. The findings have great implications on guidelines and public policy on quality improvement in cancer care. Future studies are recommended to investigate the impact of waiting time on survival in resource-constrained settings.
